# Insights Into How mHealth Applications Could Be Introduced Into Standard Hypertension Care in Germany: Qualitative Study With German Cardiologists and General Practitioners

**DOI:** 10.2196/56666

**Published:** 2025-03-28

**Authors:** Susann May, Frances Seifert, Dunja Bruch, Martin Heinze, Sebastian Spethmann, Felix Muehlensiepen

**Affiliations:** 1 Center for Health Services Research Brandenburg Medical School Theodor Fontane Rüdersdorf Germany; 2 Department of Cardiovascular Surgery, Heart Center Brandenburg Faculty of Health Sciences Brandenburg Medical School Theodor Fontane Bernau bei Berlin Germany; 3 Department of Psychiatry and Psychotherapy Immanuel Klinik Rüdersdorf, University Hospital of Brandenburg Medical School Theodor Fontane Rüdersdorf Germany; 4 Deutsches Herzzentrum der Charité – Department of Cardiology, Angiology and Intensive Care Medicine Berlin Germany; 5 Charité – Universitätsmedizin Berlin, corporate member of Freie Universität Berlin and Humboldt-Universität zu Berlin Berlin Germany; 6 AGEIS Université Grenoble-Alpes Grenoble France

**Keywords:** hypertension, mHealth apps, physicians, qualitative study, digitalization, app, application, Germany, blood pressure, cardiologists, thematic analysis, general practitioner, mobile phone

## Abstract

**Background:**

Mobile health (mHealth) apps provide innovative solutions for improving treatment adherence, facilitating lifestyle modifications, and optimizing blood pressure control in patients with hypertension. Despite their potential benefits, the adoption and recommendation of mHealth apps by physicians in Germany remain limited. This reluctance may be due to a lack of understanding of the factors influencing physicians’ willingness to incorporate these digital tools into routine clinical practice. Understanding these factors is crucial for fostering greater integration of mHealth apps in hypertension care.

**Objective:**

The aim of this study was to explore the relationship between physicians’ information needs and acceptance factors, and how these elements can support the effective integration of mHealth apps into daily medical routines.

**Methods:**

We conducted a qualitative study involving 24 semistructured telephone interviews with physicians, including 14 cardiologists and 10 general practitioners, who are involved in the treatment of hypertensive patients. Participants were selected through purposive sampling to ensure a diverse range of perspectives. Thematic analysis was conducted using MAXQDA software (Verbi GmbH) to identify key themes and subthemes related to the acceptance and use of mHealth apps.

**Results:**

The analysis revealed significant variability in physicians’ information needs regarding mHealth apps, particularly concerning their functionalities, clinical benefits, and potential impact on patient outcomes. These informational gaps play a critical role in determining whether physicians are willing to recommend mHealth apps to their patients. Key determinants influencing acceptance were identified, including the availability of robust knowledge about the apps, high-quality and reliable data, generational shifts within the medical profession, solid evidence supporting the effectiveness of the mHealth apps, and clearly defined areas of application and responsibilities within the physician-patient relationship. The study found that acceptance of mHealth apps could be significantly increased through targeted educational initiatives, enhanced data quality, and better integration of these tools into existing clinical workflows. Furthermore, younger physicians, more familiar with digital technologies, demonstrated greater openness to using mHealth apps, suggesting that generational changes may drive future increases in adoption.

**Conclusions:**

The successful integration of mHealth apps into hypertension management requires a multifaceted approach that addresses both the informational and practical concerns of physicians. By disseminating comprehensive knowledge about the variety, functionality, and proven efficacy of hypertension-related mHealth apps, health care providers can be better equipped to use these tools effectively. This approach necessitates the implementation of various knowledge transfer strategies, such as targeted training programs, peer learning opportunities, and active engagement with digital health technologies. As physicians become more informed and confident in the use of mHealth apps, their acceptance and recommendation of these tools are likely to increase, leading to more widespread adoption. Overcoming current barriers related to information deficits and data quality is essential for ensuring that mHealth apps are optimally used in routine hypertension care, ultimately improving patient outcomes and enhancing the overall quality of care.

**Trial Registration:**

German Clinical Trials Register DRKS00029761; https://drks.de/search/de/trial/DRKS00029761

**International Registered Report Identifier (IRRID):**

RR2-10.3389/fcvm.2022.1089968

## Introduction

Advancing digitalization of health care has a transformative impact on the prevention and treatment of diseases, particularly in the realm of hypertension. In this context, mobile health (mHealth) apps are increasingly gaining significance, offering innovative means for monitoring, maintaining, and preventing high blood pressure [1-3].

Hypertension poses a substantial health challenge, affecting millions of people worldwide [4]. In Germany, it is one of the most prevalent health conditions, affecting a considerable portion of the population. In Germany, 19 million people with statutory health insurance (26.3%) were diagnosed with high blood pressure in 2018 [5]. Prevention and successful management of hypertension are imperative in averting potentially severe complications, including ischemic heart disease, stroke, and renal disorders. In addition, these measures play a pivotal role in enhancing the overall quality of life of patients with hypertension [6-8].

Preventing high blood pressure using a smartphone app that can be accessed at any time appears to be a promising tool [9]. Lifestyle interventions for preventive behavior, such as improving medication adherence, restricting food intake, or promoting activity, can generally be encouraged with the help of mHealth apps [10-17]. A systematic review concluded that the use of mHealth apps in hypertension care by general practitioners could be very useful, although various barriers, such as generational differences in willingness to use digital devices and the lack of knowledge of available mHealth apps, need to be overcome [18]. In the future, an increasing number of digital technologies will be used in health care [19]. To address this development, it is necessary to understand the current health care situation, especially the insights of physicians and their perspectives on benefits and challenges, as well as their information needs, to best integrate digital technologies into care.

To date, there has been limited research on physicians’ perceptions of mHealth apps and devices and their willingness to prescribe them for hypertension care. In general, research has shown that physician adoption of mHealth apps depends on several factors, including perceived usefulness, ease of use, and trustworthiness of the technology. For example, Gagnon et al [20] found that the acceptance of mHealth apps by health care providers was highly dependent on their perception of clinical usefulness and ease of use. An Australian study highlighted concerns, including the digital divide between generations, lack of knowledge about prescription mHealth apps and devices, additional working hours, and data security concerns [21]. A French study [22] investigated general practitioners’ attitudes toward prescribing mHealth apps and identified three main attitudes among general practitioners: (1) digital engagement, emphasizing the benefits of mHealth as a complementary tool for medical practice; (2) patient protection, focusing on concerns about data security, misinformation, and patient safety; and (3) doctor protection, highlighting concerns about increased workload and the impact on the doctor-patient relationship. A recent study on attitudes toward the use of digital health applications (in German: digitale Gesundheitsanwendungen, DiGA; digital therapeutics that can be prescribed by physicians in Germany) in Germany revealed information deficits. Both doctors and patients are insufficiently informed about mHealth apps and their health benefits, leading to a lack of trust [23]. A descriptive study of German general practitioners found that 60% of general practitioners recognized mHealth apps as beneficial for the health management of their patients, but only 36% had an overall positive opinion, and only 18% regularly recommended such mHealth apps [24]. However, despite these findings regarding diverse information needs, there is little evidence specifically examining the acceptance of mHealth apps by physicians in the context of hypertension management in Germany. Although physicians generally have a positive perception of such apps, this has not yet translated into widespread adoption in practice. This discrepancy suggests the presence of underlying, unaddressed barriers—whether related to information gaps, workflow integration issues, or confidence in the technology—which prevents clinicians from actively recommending or using these tools. It is these hidden factors that our research aims to uncover and address in order to gain a deeper understanding of the dynamics influencing the adoption of mHealth apps in hypertension care. Most previous studies have either focused on other chronic diseases or on the patient’s perspective, leaving a gap in understanding physician-specific barriers related to hypertension management.

In addition, the German health care system has specific structural and regulatory characteristics that can affect the integration of mHealth apps. Germany is characterized by strict data protection regulations, as laid down in the European Union’s General Data Protection Regulation (in German: Datenschutz-Grundverordnung, DSGVO). Especially in the sensitive context of mHealth apps, this leads to additional uncertainties and reservations among physicians who want to integrate these technologies into their clinical practice. Furthermore, fragmented digital infrastructures, complex reimbursement mechanisms for digital health applications, and varying degrees of digitalization in the different health care sectors further complicate acceptance. It is crucial to address these barriers by exploring the specific information needs of physicians regarding mHealth apps to ensure their effective implementation. Evidence shows that eHealth implementations often fail because physicians are not adequately involved in the development and integration processes [25]. These structural and informational gaps contribute to the disconnect between the theoretical openness toward mHealth apps and their practical adoption in routine care.

A comprehensive understanding of physicians’ concerns, expectations, and knowledge gaps could guide the development of training programs that enhance their skills in handling digital preventive tools. Furthermore, investigating the acceptance of these technologies provides insights into the potential challenges that medical staff may face, enabling the design of user-friendly mHealth apps that can seamlessly integrate into clinical practice.

This study focused on investigating the information needs and acceptability of mHealth apps for the prevention of hypertension by health care providers. The aim of this study was to develop an understanding of how information needs and acceptance factors are related to the best integration of mHealth apps into hypertension care. This study addressed the following research questions: (1) What is the self-perceived acceptability of mHealth apps in hypertension care by physicians, and how could it be increased? (2) What information needs do physicians have, and how can these be met? (3) What is needed to integrate mHealth apps into the health care system in the long term, and what practical implications can be derived from this?

## Methods

### Study Design

This paper presents an investigation of physicians’ perspectives on the use of digital technologies in preventing hypertension. Using qualitative data collection methods, comprehensive interviews were conducted with general practitioners and cardiologists.

### Ethical Considerations

All the ethical issues were addressed. All experimental protocols were approved by the institutional or licensing committee. All methods were performed in accordance with relevant guidelines and regulations. Written consent was obtained from all participants after they were given the opportunity to ask clarifying questions. This study was approved by the Ethics Committee of the Brandenburg Medical School Theodor Fontane (E-02-20220620). Participants were informed verbally and in writing about the purpose, the procedure, the significance of the study, and the benefits and risks that may be associated with it and had the opportunity to ask questions. They were also informed that they had the right to withdraw their consent to participate in the study at any time, either verbally or in writing, without giving reasons. They were also informed that personal data would be recorded and stored, whereby the data would be pseudonymized, but that no data would be published that could be used to identify the person. For security reasons, the data received from the participants was always stored in a password-protected folder on a secure desktop computer. Written consent was obtained after participants were given the opportunity to ask questions. Patients were not involved in designing this study. Participants were offered €75 (approximately US $77.89) as an incentive for their participation in the study.

### Participant Recruitment

The participants were selected using purposive sampling [26]. We included both general practitioners and cardiologists. Further inclusion criteria were current involvement in the treatment of patients with hypertension and interest in participating in an interview. The participants were recruited by snowball sampling. The following sources were used for the recruitment:

- Social media accounts of the university (Brandenburg Medical School Theodor Fontane): Facebook, Instagram, and Twitter.
- Association of Statutory Health Insurance Physicians Brandenburg: newsletter.
- General Practitioners Association Brandenburg: information study participation at events.
- Personal contact among colleagues.

Physicians were offered €75 (US $77.89) as an incentive for their participation in the study, which was financed from the earmarked research funds. This measure was taken to reward the time spent by doctors and to ensure sufficient willingness to participate. This study was conducted independently and was not directly related to the Innovation Committee of the Federal Joint Committee (G-BA: Committee in Germany that determines which medical services are provided to people with statutory health insurance). There were no external influences on the design, conduct, or evaluation of the study, and there were no conflicts of interest that could compromise the independence and reliability of the results.

### Data Collection

A preliminary semistructured interview guide was collaboratively developed by a multiprofessional team (SM, FM, DB, and SS). The interview guide comprised open-ended questions exploring participants’ familiarity with digital applications, particularly mHealth apps, their perceived benefits and barriers, their information needs for prevention, and aspects that influence their acceptance of mHealth apps. Sample questions included inquiries about the use of mHealth apps in hypertension treatment, perceived benefits, encountered barriers, and level of knowledge about mHealth apps (please see the interview guide in Multimedia Appendix 1). In addition, sociodemographic data, such as profession, gender, age, number of inhabitants, duration of professional activity, and setting, were collected. The present findings report on information needs and aspects of acceptance.

To ensure the clarity and relevance of the questions, a pilot test of the interview guide was conducted with 5 eligible participants recruited from clinics and outpatient physicians in the study’s catchment area. All interviews were conducted through telephone between October 2022 and March 2023 and were recorded and transcribed verbatim. Data collection continued until no new findings emerged and content saturation was achieved. Saturation was defined as code saturation, indicating no additional issues identified, and meaning saturation, indicating no further dimensions, nuances, or insights [27]. Field notes were recorded for each interview. They were used to understand the interview situation better.

### Data Analysis

Qualitative analysis of the interviews was performed iteratively by SM and FM based on thematic analysis [19] using MAXQDA Analytics Pro 2022 (release 22.1.0; Verbi GmbH). After transcription of the audio material, the analysis began with familiarization with the interviews. A sample of the transcripts was then independently analyzed using SM and FM to iterate and finalize a comprehensive codebook. The main categories and subcategories are formed from these codes. The thematic analysis included reading each transcript, identifying patterns, assigning codes, and formulating themes and subthemes from the data. After this process, the data were checked, compared, and discussed to eliminate discrepancies [28]. If no new data, themes, or relationships could be identified, data saturation was achieved [29]. To present these findings, significant excerpts from the transcriptions were chosen as representative quotes. These quotes have been translated into English and incorporated into the manuscript. The manuscript was compiled in accordance with the COREQ (Consolidated Criteria for Reporting Qualitative Research; see Multimedia Appendix 2) [30].

## Results

### Overview

A total of 24 interviews were conducted and analyzed. The mean duration of the interviews was 42 (SD 5.75; minimum 33, maximum 56) minutes. The mean age of the participants was 50 (range 35-74) years. Most participants were male (13/24, 54%). In total, 14 cardiologists and 10 general practitioners participated in the study. A total of 7 individuals worked in the inpatient setting, and 18 people worked in the outpatient setting. Furthermore, 1 person worked in both settings. In addition, 4 of the physicians already had experience with digital applications in daily practice or were working with them. Table 1 shows the detailed characteristics of the study participants.

**Table 1:** Detailed characteristics of study participants.

ID	Profession	Duration of professional activity (years)	Age (years)	Sex	Number of inhabitants (workplace), n	Setting
K1	Cardiologist	14	45	Male	3.6 million	Inpatient
K2	Cardiologist	49	74	Male	1.4 million	Outpatient
K3	Cardiologist	7	48	Male	60,000	Outpatient and inpatient
K4	Cardiologist	20	57	Male	10,000	Outpatient
K5	Cardiologist	8	44	Male	310,000	Outpatient
K6	Cardiologist	29	62	Male	3.4 million	Inpatient
K7	Cardiologist	38	55	Female	90,000	Outpatient
K8	Cardiologist	4	41	Female	3.4 million	Inpatient
K9	Cardiologist	18	55	Female	20,000	Outpatient
K10	Cardiologist	7	41	Male	30,000	Outpatient
K11	Cardiologist	7	40	Female	13,000	Outpatient
K12	Cardiologist	11	52	Male	300,000	Outpatient
K13	Cardiologist	20	51	Female	130,000	Inpatient
K14	Cardiologist	20	51	Male	3.6 million	Inpatient
H1	General practitioner	21	45	Male	3.6 million	Outpatient
H2	General practitioner	29	57	Female	15,000	Outpatient
H3	General practitioner	5	40	Male	70,000	Outpatient
H4	General practitioner	5	40	Female	70,000	Outpatient
H5	General practitioner	30	60	Female	3.6 million	Outpatient
H6	General practitioner	4	35	Female	3.6 million	Outpatient
H7	General practitioner	5	56	Male	8000	Outpatient
H8	General practitioner	12	36	Male	7000	Inpatient
H9	General practitioner	30	63	Female	900	Outpatient
H10	General practitioner	21	54	Female	3.6 million	Outpatient

### Self-Perceived Acceptability and Acceptance-Increasing Factors

Neither a particularly positive nor negative attitude toward mHealth apps was identified. Rather, physicians’ response behavior is characterized by heterogeneity. mHealth apps are generally accepted by physicians, as long as they are not part of their daily medical routines. This means that they are certainly open-minded but not in their own care settings, especially due to a lack of experience in using mHealth apps.

Well, I have to answer that in such a way that it is neutral, so to speak, because I simply do not have enough experience so far.

K3

I do not think I need it in my daily routine. I know a bit about high blood pressure and about general measures and therapies, and I try to convey that to the patients in conversation and then use it. So, I do not think I have discovered the need for it at the moment, to what extent it could take work off my hands or to what extent the patients could be better blood pressure controlled, or something else.

K3

**Figure 1:**
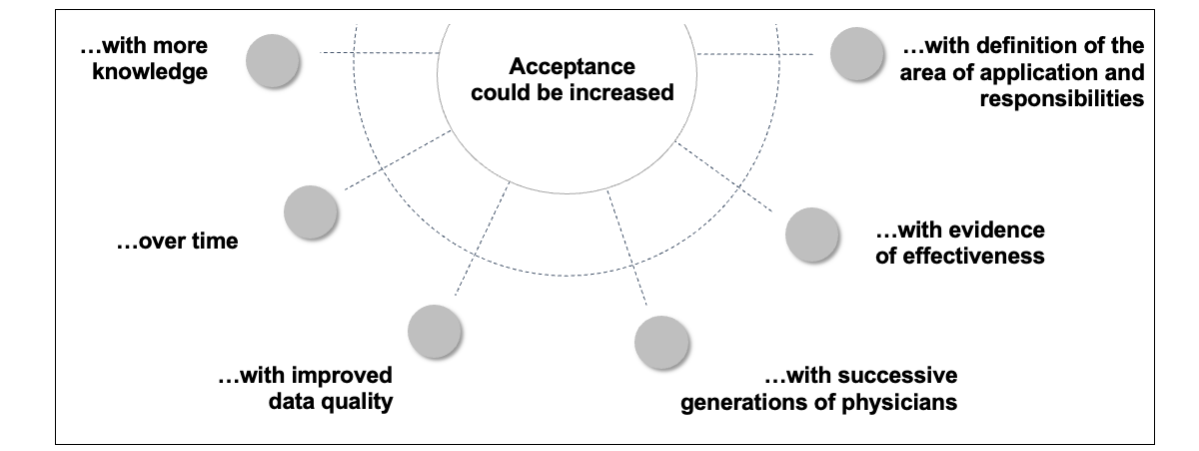
Identified subthemes of the acceptance-increasing factors (coding tree).

Nevertheless, physicians emphasized that acceptance could increase. Figure 1 shows the factors associated with the increase in acceptance, and are further discussed in the following subsections.

#### Acceptance Could Be Increased With More Knowledge

Overall, the data revealed that physicians’ acceptance of hypertension apps could increase with a deeper understanding of their benefits, targeted training, and seamless integration into existing clinical workflows.

And these reservations are simply ignorance and also ignorance of the actual tools that are available.

K2

#### Acceptance Could Be Increased Over Time

The interviewed physicians emphasized that the acceptance of digital technologies and mHealth apps in medical practice is often subject to a natural maturation process in which initial concerns and uncertainties diminish over time. This also applies to hypertension apps, which serve as technologies for the monitoring and self-management of blood pressure. For example, physicians indicated that increasing acceptance is significantly influenced by time as experience accumulates, research findings grow, and technology proves its reliability. In the early stages of mHealth app adoption, some physicians may be hesitant due to uncertainties about data accuracy, privacy concerns, and integration into existing workflows. However, over time, positive experiences and feedback from colleagues who have already implemented such technologies will help alleviate concerns and build confidence in the effectiveness of mHealth apps.

It will be unavoidable (the use of apps in daily routines). Thus, the industry is flooding us with apps. Studies are certainly required to be conducted. That's all true. We will certainly be doing a lot of things digitally in ten or 20 years.

K14

#### Acceptance Could Be Increased With Improved Data Quality

A key factor influencing the acceptance of hypertension apps among physicians is the data quality offered by these apps. Physicians reported concerns regarding the reliability of the collected data, leading to reluctance to rely on such technologies. In particular, it is not always guaranteed that patients enter their blood pressure data correctly. Overall, the ongoing improvement in data quality will help reduce existing reservations about hypertension apps and further increase their acceptance among physicians.

I believe that we can foresee that this will be accepted because the data [on use and effectiveness] will presumably also have better quality in the future.

K2

#### Acceptance Could Be Increased With Successive Generations of Physicians

The ongoing generational shift in the medical profession plays a decisive role in increasing acceptance of hypertension apps. Physicians emphasize that younger physicians who have grown up in an era of digital innovation are often more open to the use of modern technology in medical practice. Unlike the older generations, who may be more familiar with traditional methods of patient monitoring, young physicians already have an affinity for digital health solutions. Digital natives recognize the potential of mHealth apps as efficient technologies for improving patient care. Their willingness to accept and integrate innovative technologies is fueled by the realization that these apps can not only promote patient autonomy but also increase efficiency in clinical practice. The younger generation’s openness to new technologies is driving the integration of mHealth apps into the modern medical landscape. As the digital-savvy generation enters medical practice, the use of mHealth apps is likely to be seen as a natural part of treatment. Interestingly, participants in all age groups expressed this impression.

Of course, this [use of mHealth apps] comes with younger physicians who have grown digitally in a completely different way. For them, it will be a matter of course that they send their data, laboratory results, and everything else digitally.

K2

#### Acceptance Could Be Increased With Evidence of Effectiveness

The physicians reported that they need solid evidence that the use of hypertension apps is not only technologically advanced but also achieves positive results in clinical practice. Clear evidence of improvements in blood pressure control, reduction of complications, and overall better health care is essential from the point of view of strengthening physician conviction.

Certainly, scientific proof that it makes sense. I am always in favor of prescribing something like this if it is proven to be beneficial for the patient. However, I do not see that yet.

K9

#### Acceptance Could Be Increased With a Definition of the Area of Application and Responsibilities

In the interviews, the physicians addressed the fact that the purpose and functions of the use of mHealth apps are often not defined, either for the patients themselves or within physician-patient communication. Physicians point out that it should be clear to both patients and physicians how an mHealth app is integrated into care. Whether the mHealth app is used as a lifestyle product or a monitoring tool. Whether physicians are actively involved in the evaluation of the data, whether they should take on a controlling function, and whether the use of the mHealth app is only for observation or should even have consequences, for example, through advice from the physician.

I always think the question is interesting: what do I want to achieve with the app? Do I want truest for therapy management? These are the patients we were just talking about, who are not health-conscious per se. Do we want people to feel comfortable with their illness and feel that they are doing an incredible amount for themselves? That brings us to advice, offers, and lifestyle apps - that is, what I call it, right? Somewhere along the line, if it is from a medical context, it should take both facets into account but focus more on the medical context.

K14

### Information Needs

**Figure 2:**
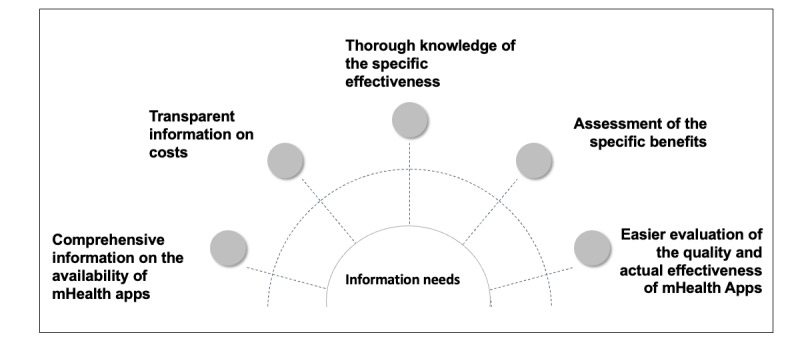
Identified subthemes of the information needs (coding tree). mHealth: mobile health.

A variety of information needs became apparent in the interviews. Figure 2 shows the subthemes of information needs.

#### Comprehensive information on the Availability of mHealth Apps

Physicians are generally interested in using mHealth apps in their daily routine. However, there is a lack of information on the range of mHealth apps that can be integrated into hypertension care. There is a knowledge gap regarding the functions, data security, accuracy, and clinical benefits of hypertension apps.

In my opinion, the market has been overly flooded. And then you do not even know which apps are actually certified and which are available. Thus, we cannot know all apps that are buzzing around the market.

K7

#### Transparent Information on Costs

A challenging aspect in the context of integrating mHealth apps into medical practice is that many physicians are often not fully informed about the costs associated with these mHealth apps. Nontransparent pricing and diverse pricing models of various providers lead to uncertainty. This lack of clarity regarding financial aspects can affect physicians’ decision-making and even make them reluctant to integrate such mHealth apps into their treatment plans.

I do not know enough about what is available or what you can choose from and what that means in terms of costs.

K3

#### Thorough Knowledge of the Specific Effectiveness

The lack of knowledge regarding the specific effectiveness of hypertension apps poses a further challenge for physicians. There is often a lack of clear information about how these apps can have a positive effect on the treatment of patients with high blood pressure in practice. In contrast, physicians emphasize that they know and can assess the use and effects of medication. Physicians need detailed insights into the algorithms, data analysis methods, and interpretation of the information generated by the mHealth apps to understand and assess their effectiveness.

Yes, and I mean, when I prescribe ramipril, it's because I know this medication, I know its effects, side effects, I have used it for a long time, and I know it. However, these apps are expensive. They cost 500 euros, so I want to know in advance what I am prescribing and how they work.

H10

#### Assessment of the Specific Benefits

The limitations in assessing the specific benefits of hypertension apps pose a challenge to physicians. There is often a lack of clear and convincing information about how these mHealth apps actually benefit patients. In addition, physicians are unable to assess the extent to which apps benefit their daily practice.

So, I think at the moment I just have not identified the need for myself to what extent this could take work off my hands, how it would benefit the patients, and to what extent the patients could be better blood pressure adjusted or something.

K3

#### Easier Evaluation of the Quality and Actual Effectiveness of mHealth Apps

Physicians’ difficulty in assessing the quality of mHealth apps is a key barrier. There is often a lack of clear benchmarks and objective criteria for evaluating the actual benefits of these mHealth apps for individual patient care. The lack of standardized evaluation methods makes it difficult for physicians to determine the degree of effectiveness of a particular hypertension app and assess its clinical relevance.

And I would always look at the study situation first because many of them have only been proven with very poor studies and are, therefore, only provisionally approved. I do not think I would prescribe them at the moment because I think they cost money and have not been sufficiently tested. I do not even know how good they are.

H6

Evaluating the effectiveness of hypertension apps is a complex challenge for physicians. The lack of standardized criteria for collecting outcome data and comprehensive clinical trials makes it difficult to draw objective conclusions about the effectiveness of these mHealth apps. Physicians often struggle to assess how well hypertension apps actually work in practice and what concrete contribution they can make to improving blood pressure control and the health of their patients. To make matters worse, many of the available hypertension apps have different functions and approaches, which further complicate a standardized assessment.

As I said, I’m too uneducated when it comes to this. I am unfamiliar with any studies and do not know how they work or how effective they are.

K3

## Discussion

### Principal Findings

This study provides new insights into the factors influencing physicians’ adoption of mHealth apps in the context of hypertension management in Germany. Our findings reveal a complex interaction of factors—from information gaps and workflow integration challenges to uncertainties around data security and reimbursement structures—which influence physicians’ willingness to integrate these technologies into their daily routines.

This study provides a nuanced view of the acceptance of mHealth apps among cardiologists and general practitioners, particularly in the context of hypertension management. While there is a general openness to digital technologies among physicians, hesitation persists, primarily due to a lack of familiarity and experience with these technologies. A significant barrier to the adoption of mHealth apps is a limited understanding of their benefits and functionalities. Physicians have indicated that targeted training could greatly enhance acceptance. Over time, acceptance is expected to increase as experience grows and positive outcomes are observed. However, concerns regarding the accuracy of patient-entered data remain a major issue, with improved data reliability being essential for building trust in these technologies. Younger physicians, who are more familiar with digital technologies, tend to be more receptive to mHealth apps, suggesting that acceptance is likely to grow as the medical field evolves. Nevertheless, substantial information gaps, including a lack of awareness of the range and benefits of available apps, hinder adoption. In addition, unclear cost structures and the absence of standardized methods for assessing quality and effectiveness further contribute to physicians’ reluctance to recommend and use these technologies.

### Comparison With Previous Work and Theoretical Embedding

#### Self-Perceived Acceptability of Physicians Toward mHealth Apps in Hypertension Care

A major obstacle to the introduction of mHealth apps into standard hypertension care is a lack of understanding of their benefits and functions. The aspect “Acceptance could be increased with more knowledge” can be linked to the theoretical concept of the “Technology Acceptance Model” (TAM). TAM, developed by Davis [31], postulates that the acceptance of technology depends largely on 2 factors: its perceived usefulness and perceived ease of use. In terms of mHealth apps, physicians with a sound understanding of the specific benefits and ease of integration of these technologies into their clinical practice are more likely to accept and use them. When physicians are better informed about the positive clinical outcomes that can be achieved through mHealth apps, as well as aspects related to practical application, such as data integrity and privacy, their perceived usefulness and ease of use increase. This increased knowledge reduces uncertainty and skepticism, which ultimately leads to greater acceptance and integration of technology into everyday medical practice. A systematic review examining factors influencing acceptance concluded that the lack of routine use of mHealth apps is primarily due to insufficient knowledge and information regarding their integration into daily routines. [32]. The need for information is a key component of acceptance, particularly in telemedicine [33], as well as in mHealth apps. The way in which information is provided and how well users’ information needs are met have a significant influence on their willingness to accept and use a particular technology. A recent study [34] pointed out that the level of awareness of mHealth apps for hypertension should be increased through targeted information campaigns and physician training. In addition, physicians need to test mHealth apps, and experience and acceptance would increase.

Acceptance is expected to increase over time as more experience is gained and positive outcomes are observed. This aspect can be linked to the diffusion of innovation theory proposed by Rogers [35]. According to Rogers’ theory, the adoption of new technologies follows a specific pattern over time and is categorized into 5 stages: innovators, early adopters, the early majority, the late majority, and laggards. Initially, only a small group of innovators and early adopters might embrace mHealth apps; however, as more physicians witness positive outcomes and share their experiences, the early and late majority will gradually adopt these technologies. Over time, as research continues to provide robust evidence of the effectiveness and reliability of mHealth apps and as the technology becomes more integrated into standard medical practices, the remaining skeptical physicians (the laggards) will also start to accept and use these technologies. The stronger the habit of using mHealth apps, the stronger the connection between user satisfaction and willingness to continue using the apps [36]. People who are satisfied with and develop habits related to new technologies are more likely to use these apps in the long term [36].

Another important finding of our study is that there are concerns about the accuracy of the data entered by patients, and improved data reliability is crucial for building trust in these technologies. This is in line with the Unified Theory of Acceptance and Use of Technology 2 (UTAUT2) [37]. One of the key determinants within this framework is the concept of trust, which is strongly influenced by the quality of the data provided by technology. High-quality data, characterized by accuracy, reliability, timeliness, and relevance, can significantly increase physician trust in mHealth apps. When data quality is assured, physicians are more likely to perceive technology as useful and reliable, reducing their uncertainty and increasing their willingness to adopt and integrate these technologies into their clinical practice. This improved confidence and the perceived benefits of better data quality can lead to higher overall adoption of mHealth apps among healthcare professionals. A recent study [38] showed that doctors were willing to use mHealth apps if they felt comfortable and safe. This highlights the importance of user experience in the adoption of mHealth apps by health care professionals. The intention to use mHealth apps in everyday health care depends on the perceived reliability of these technologies. If physicians perceive mHealth apps as reliable and trustworthy, they are more likely to regularly integrate them into their workflows [36].

The identified aspect, “Acceptance could be increased with successive generations of physicians,” can be linked to the concept of the “Digital Divide,” which highlights the disparities in access to and use of digital technologies across different demographic groups [39-41]. Younger physicians with digital technologies are less likely to experience the barriers associated with the digital divide. They generally have better access to digital technologies, higher levels of digital literacy, and greater confidence in using technology in medical practice. As these younger, more technologically adept physicians enter the workforce, acceptance and integration of mHealth apps will naturally increase. Several studies have shown that younger doctors are more likely to use mHealth apps, and in an Australian study, both patients and physicians cited age as a barrier to mHealth app prescription but also provided examples of exceptions to this age-based divide in digital readiness [21]. A German study also identified a trend in physician age. Younger physicians are often more open to digitalization and welcome innovations such as digital health applications [42]. Another study from Germany specifies the age aspect and shows that the highest acceptance rates of digital health applications are expected in the 30-49 years age group [43].

#### Physicians’ Information Needs and How to Address Them

Based on the assumption that acceptance could be increased with more knowledge, it is particularly important to determine what information needs exist in the context of mHealth apps in hypertension care. The interviewed physicians expressed a high demand for information regarding mHealth apps for hypertension care and various information deficits. At the same time, however, there are reservations due to uncertainties regarding effectiveness, data security, and integration into the clinical workflow. A thorough and, at the same time, a low-threshold explanation of the benefits and security aspects of such applications is therefore crucial to increase acceptance of mHealth.

The lack of information also explains why mHealth apps are not yet integrated into the health care infrastructure. Few specialists currently use or recommend mHealth apps in their daily routines [44]. This leads to a discrepancy between the available digital technologies, such as mHealth apps, and their actual use in clinical practice. The gap between the daily work with patients and the availability of mHealth apps is becoming increasingly apparent, and in this context, the “digital divide,” which refers to differences in access, use, and understanding of digital technologies in health care, is also prevalent among health care practitioners [40]. On the one hand, there is the daily interaction with patients, which is characterized by personal contact, traditional diagnostic methods, and conventional treatment approaches. On the other hand, a growing number of mHealth apps offer digital solutions for monitoring health parameters, managing diseases, and accessing health information. Our results show that physicians do not know what mHealth apps can do, what it means to use them, or how much effort is involved.

In addition, a lack of information regarding costs also contributes to this. Reviews demonstrate that physicians are less willing to use eHealth technologies if the reimbursement is not clearly defined or communicated transparently [45,46]. This uncertainty surrounding financial compensation not only hinders the adoption of mHealth apps but also undermines their potential integration into routine clinical practice. Clear guidelines and transparent communication regarding reimbursement policies are essential to enhance healthcare providers’ confidence in adopting these technologies.

An approach to closing the information gap resulting from the lack of assessment of the quality and effectiveness of mHealth apps is to use assessment tools. Various studies have already examined this and concluded that quality assessment tools are a viable approach to distinguish high-quality mHealth apps from lower-quality mHealth apps [47,48]. By implementing standardized evaluation criteria, these tools can provide health care professionals with the necessary insights to make informed decisions about which apps should be integrated into their practice, ultimately enhancing patient care. Establishing clear and transparent evaluation metrics covering aspects such as design, information/content, usability, functionality, ethical issues, security and privacy, and user-perceived value would not only build trust among health care providers but also facilitate broader acceptance and integration into routine care [47].

### Integrating mHealth Apps Into the Health Care System: Practical Implications

Based on our findings, we derived 4 fields of action (Multimedia Appendix 3) to achieve a more successful implementation of mHealth apps in physicians’ daily routines.

### Definition of Areas of Application of Responsibilities

Before mHealth apps are used for hypertension treatment, their functions should be specified in advance. The physician and patient should discuss the area of application, therapy goal, and type of collaboration. If the mHealth app is used as a lifestyle product, the patient tracks their parameters alone, and the physician is not directly involved in medical treatment. This means that the physician has no control, and there are no responsibilities for the physician; that is, they do not have to actively intervene. If the mHealth app is used as medical advice, the patient and physician monitor the parameters together, and the effectiveness and benefits should be proven in advance. This implies that physicians are directly involved in medical treatment. The physician and the patient discuss the parameters together, and the physician assumes a certain degree of control and can actively intervene. This approach could lead to mHealth apps being better accepted by physicians as medical advice and used by them more frequently. Multimedia Appendix 4 illustrates the proposed approach.

### Specific Recommendations for Integration Into Daily Routines With Guidelines and Options for Measuring Outcomes

A consistent methodology for collecting outcome data and establishing evidence-based criteria is essential to enable clinicians to better understand the performance of these applications. Creating clear guidelines and evaluation tools will help reduce uncertainty about their effectiveness and provide physicians with a solid foundation for making decisions about integrating hypertension apps into their practice. In addition, interfaces and clear guidelines for the integration of such technologies must be created to promote their use by physicians. Intensive efforts should be made to obtain more information about the range, effects, and modes of action of mHealth apps [34].

### Information Campaigns

In health care, knowledge translation plays a key role in the integration of new medical technologies into everyday care [49]. The acceptance of new technologies depends heavily on how well physicians understand the underlying scientific evidence, how effectively these are implemented in clinical practice, and how they bridge the gap between research and practice. Knowledge translation is essential as it facilitates the dissemination of evidence-based practices, ensuring that the latest research findings and medical innovations reach health care practitioners, policymakers, and, most importantly, the general public. Information campaigns serve as powerful vehicles for knowledge translation, acting as conduits for conveying complex medical information in an accessible and understandable manner. As the acceptance of mHealth apps is also generation-dependent, strategies that appeal to both younger and more experienced physicians should be developed. This can be achieved through various approaches, such as peer learning or mentoring. Furthermore, there should be special training programs for physicians that provide detailed information about the available mHealth apps, their functions, and scientific evidence for their effectiveness [22].

### Active Use of the mHealth Apps in Collaboration With the Developers

For physicians, actively engaging with and experimenting with these apps is both practical and crucial. By exploring digital technologies, such as mHealth apps, physicians can gain valuable insights into their functions, usability, and potential benefits [50]. By actively using health apps, physicians can develop a nuanced understanding of how these technologies operate in real-world medical settings. This hands-on experience not only equips physicians with the knowledge to recommend suitable apps to patients but also fosters a deeper appreciation of the role these technologies can play in modern health care. Furthermore, physicians acting as early adopters can contribute significantly to the overall acceptance and integration of health apps within the medical community.

### Strengths

To the best of our knowledge, this is the first qualitative thematic analysis to provide an in-depth understanding of the acceptance and information needs related to mHealth apps for hypertension prevention. While our study specifically examined physicians’ acceptance and information needs regarding mHealth apps in hypertension management, the findings suggest that many of the factors identified, such as the need for reliable data, evidence of effectiveness, and clearly defined areas of application, may also be applicable to the management of other chronic diseases such as diabetes. This potential transferability indicates that barriers and facilitators to adopting mHealth apps in medical practice share a common foundation that extends beyond individual conditions.

### Limitations

The findings could potentially be influenced by selection bias, as individuals with a greater interest in the topic might have been more inclined to participate in the study. The sampling strategy employed may not have reached a representative cross-section, leading to a lack of recording of the essential aspects. Consequently, the generalizability of the results is hindered. It should also be mentioned that the interviews were conducted over the telephone. The lack of nonverbal cues, such as body language, facial expressions, and eye contact, can make it difficult to capture the full range of emotions and reactions. Another limitation of this study is that only 4 participating physicians had experience with mHealth apps in their daily routines. This could limit the validity of the results, as the perspectives of physicians without experience using such apps could be less informative. In addition, it is important to note that the patient interviews were not conducted during this study. Including patient perspectives could have provided a more rounded understanding of the acceptance of and information needs related to mHealth apps in hypertension care. Their absence from our research presents an area for future exploration to enrich and validate our findings. In other parts of the study, in which this study was also conducted, the patients are specifically interviewed [32].

Recognizing the preliminary nature of this broad exploration into “hypertension mHealth apps,” the authors acknowledge that this serves as an initial foray. Given the extensive and diverse nature of the topic, future studies should be tailored to specific health care contexts to expand on these initial findings. These limitations present a clear avenue for future research. Plans are underway to broaden the study scope by including a larger sample in Germany [51] and to validate the qualitative outcomes through a comprehensive questionnaire survey. Subsequent investigations should focus on the identification of patients suitable or unsuitable for mHealth apps in hypertension care. Attention should also be directed toward effective strategies for educating health care professionals about mHealth and digital health in a broader sense. In addition, exploring reimbursement strategies that reinforce the optimal use of mHealth for managing chronic conditions is imperative.

Finally, the potential barriers identified here regarding the prescription of apps for hypertension by physicians pertaining to the German health care system. Further studies are required to determine whether these aspects are applicable to physicians in other countries.

### Conclusion

The use of mHealth apps in hypertension management is currently characterized by information deficits and reduced acceptance among physicians. This study underlines the need for intensive knowledge translation and transfer. The active involvement of physicians in the development and implementation of mHealth apps in hypertension care is particularly important to move from individual self-management by patients to formalized mHealth app-supported health care to prevent hypertension.

## Appendix

Multimedia Appendix 1 Interview guide.

Multimedia Appendix 2 COREQ (Consolidated Criteria for Reporting Qualitative Research) checklist.

Multimedia Appendix 3 The 4 fields of actions to achieve a more successful implementation of mobile health apps in physicians’ daily routines.

Multimedia Appendix 4 Specification of the use of mobile health apps.

## Abbreviations

COREQ: Consolidated Criteria for Reporting Qualitative Research
mHealth: mobile health
TAM: Technology Acceptance Model
UTAUT2: Unified Theory of Acceptance and Use of Technology 2

## Data Availability

The datasets generated or analyzed during this study are available from the corresponding author on reasonable request. For further questions regarding the reuse of data, please contact the corresponding author.
