# Supplementary material for: Insights Into How mHealth Applications Could Be Introduced Into Standard Hypertension Care in Germany: Qualitative Study With German Cardiologists and General Practitioners
Source: JMIR Mhealth Uhealth. 2025 Mar 28;13:e56666. doi: 10.2196/56666 (PMC11992499; doi:10.2196/56666)
Supplement: Multimedia Appendix 1 [file mhealth_v13i1e56666_app1.pdf]

Interview Guide Cardiologists & General Practitioners

<table>
<tr><td>Narrative impulse</td><td colspan="2">Specific questions</td><td>Maintenance and control questions</td></tr>
<tr><td>Could you imagine digital applications supporting you in the treatment of hypertensive patients? Tell us about it.</td><td colspan="2">-In your opinion, to what extent could m-Health apps be helpful in promoting a healthy lifestyle among patients?

-What conditions would need to be in place in order to use corresponding applications (patient and doctor-related conditions, as well as structural conditions/framework/further support)?

-What information is particularly important for you as a doctor when patients use m-Health apps?

-How should this information on the use of digital care services by patients ideally be integrated into your treatment?

-How should information ideally be communicated to patients?</td><td rowspan="2">Can you tell more about this?

And then?

What was that like for you?

How do you see it?

Can you elaborate on that, please?

Could you give an example of that, please?

What do you mean specifically?</td></tr>
<tr><td>Which m-Health apps are you familiar with in the context of hypertension treatment?</td><td>If m-Health apps are named:

-How did you become aware of m-Health apps in the context of hypertension treatment?

-Are there situations in which you think: "I actually need more</td><td>If no m-Health apps named:

-Why is it that you are not aware of any m-Health apps?

-Who would you like to receive information from?</td></tr>
</table>

	information about what is available"? Please describe.<br><br>-Who would you like to receive information from?<br><br>-How should information ideally be communicated?	-How should information ideally be communicated?	
	-Think back for a moment. Have you ever been asked about m-Health apps by your patients? What was that like? Describe it.<br><br>-How do you rate the usability of m-Health apps by your patients?<br><br>-How easy or difficult is it for your patients to use them in everyday life?<br><br>-How do you rate the effectiveness of these m-Health apps? What observations have you perhaps already made?<br><br>-In which areas can patients be particularly supported?<br><br>-How do you rate the evidence for m-Health apps?		
