# Supplementary figures and images for: Insights Into How mHealth Applications Could Be Introduced Into Standard Hypertension Care in Germany: Qualitative Study With German Cardiologists and General Practitioners
Source: JMIR Mhealth Uhealth. 2025 Mar 28;13:e56666. doi: 10.2196/56666 (PMC11992499; doi:10.2196/56666)

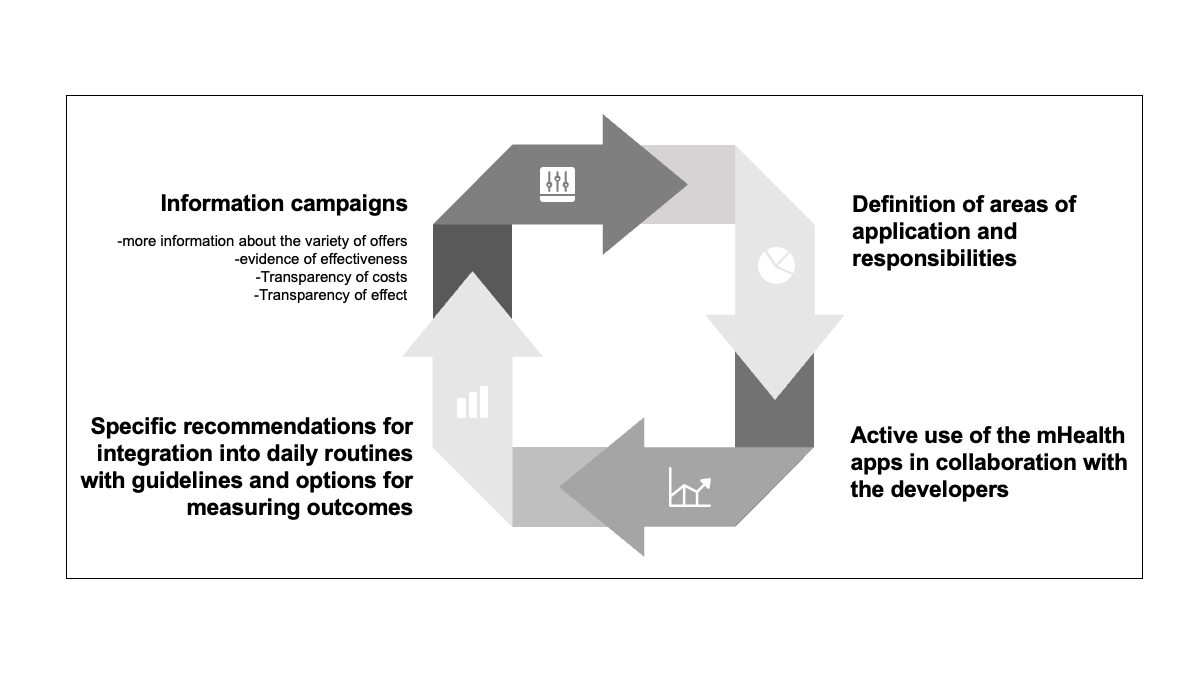

Supplement: Multimedia Appendix 3 [file mhealth_v13i1e56666_app3.png]

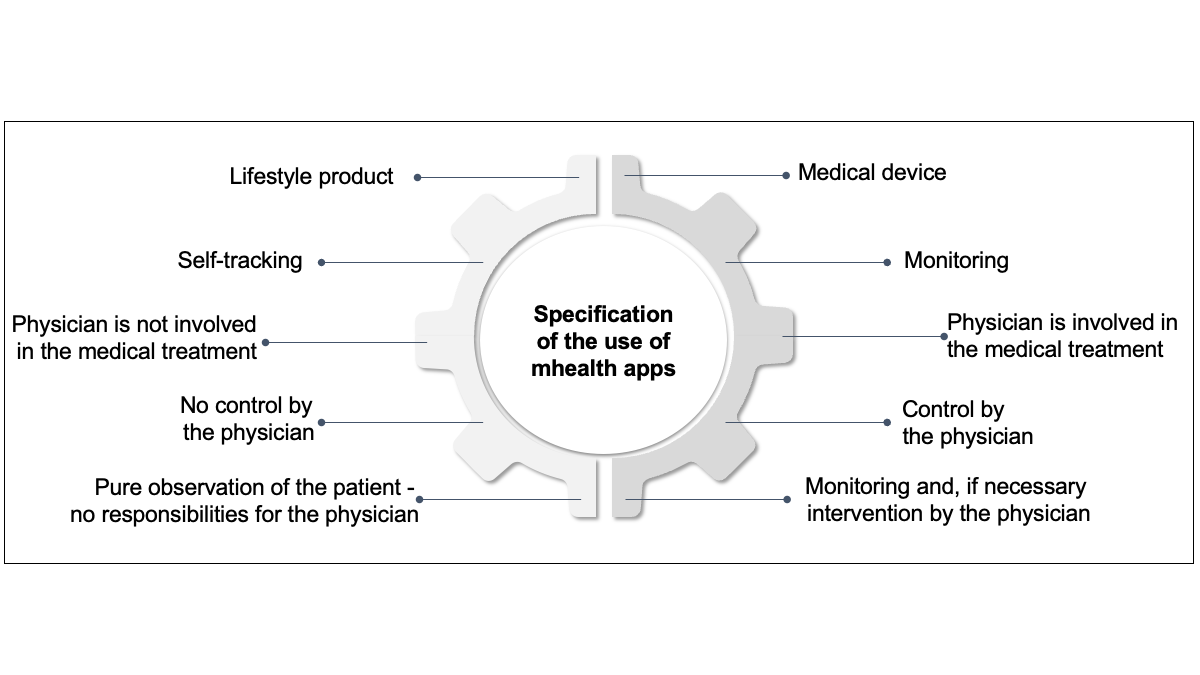

Supplement: Multimedia Appendix 4 [file mhealth_v13i1e56666_app4.png]
